# The effects of FTO gene rs9939609 polymorphism on the association between colorectal cancer and dietary intake

**DOI:** 10.3389/fnut.2023.1215559

**Published:** 2023-07-20

**Authors:** Maryam Gholamalizadeh, Mona Jonoush, Khadijeh Abbasi Mobarakeh, Arezoo Amjadi, Farkhondeh Alami, Neda Valisoltani, Seyed Ali Askarpour, Ghasem Azizi-Tabesh, Mohammad Keshavarz Mohammadian, Mohammad Esmail Akbari, Masoumeh Rajabibazl, Mahdi Alemrajabi, Jafar Poodineh, Hossein Sadeghi, Payam Hosseinzadeh, Samaneh Mirzaei Dahka, Mostafa Badeli, Seyed Alireza Mosavi Jarrahi, Saeid Doaei

**Affiliations:** ^1^Cancer Research Center, Shahid Beheshti University of Medical Sciences, Tehran, Iran; ^2^Department of Clinical Nutrition and Dietetics, Faculty of Nutrition and Food Technology, National Nutrition and Food Technology Research Institute, Shahid Beheshti University of Medical Sciences, Tehran, Iran; ^3^Food Security Research Center and Department of Community Nutrition, School of Nutrition and Food Science, Isfahan University of Medical Sciences, Isfahan, Iran; ^4^Department of Nutrition, School of Nutritional Sciences and Food Technology, Kermanshah University of Medical Sciences, Kermanshah, Iran; ^5^Student Research Committee, Department of Nutrition, Faculty of Medicine, Urmia University of Medical Sciences, Urmia, Iran; ^6^Department of Clinical Nutrition. School of Nutrition Science and Dietetics. Tehran University of Medical Sciences, Tehran, Iran; ^7^Division of Food Safety and Hygiene, Department of Environmental Health Engineering, School of Public Health, Tehran University of Medical Sciences, Tehran, Iran; ^8^Genomic Research Center, Shahid Beheshti University of Medical Sciences, Tehran, Iran; ^9^Department of Nutrition, Science and Research Branch, Islamic Azad University, Tehran, Iran; ^10^Department of Tissue Engineering and Applied Cell Sciences, School of Advanced Technologies in Medicine, Shahid Beheshti University of Medical Sciences, Tehran, Iran; ^11^Clinical Research Development Center (CRDC), Firoozgar Hospital, School of Medicine, Iran University of Medical Sciences, Zabol, Iran; ^12^Department of Clinical Biochemistry, School of Medicine, Zabol University of Medical Sciences, Tehran, Iran; ^13^Genomic Research Center, Shahid Beheshti University of Medical Sciences, Tehran, Iran; ^14^Gastrointestinal and Liver Diseases Research Center (GLDRC), Iran University of Medical Sciences, Tehran, Iran; ^15^School of Nursing and Midwifery, Guilan University of Medical Sciences, Rasht, Iran; ^16^Department of Nutrition, Urmia University of Medical Science, Urmia, Iran; ^17^Department of Social Medicine, Medical School, Shahid Beheshti University of Medical Sciences, Tehran, Iran; ^18^Department of Community Nutrition, National Nutrition and Food Technology Research Institute, Faculty of Nutrition Sciences and Food Technology, Shahid Beheshti University of Medical Sciences, Tehran, Iran

**Keywords:** colorectal cancer, FTO gene, diet, nutrient, FTO

## Abstract

**Background:**

FTO gene is associated with obesity, dietary intake, and the risk of colorectal cancer (CRC). In this study, patients with colorectal cancer were assessed for the interactions between FTO gene polymorphisms and dietary intake.

**Methods:**

This case–control study was carried out on 450 participants aged 35–70 years including 150 patients with colorectal cancer and 300 healthy controls. Blood samples were collected in order to extract DNA and genotyping of FTO gene for rs9939609 polymorphism. A validated 168-item food frequency questionnaire (FFQ) and the Nutritionist-IV software were used to assess dietary intake.

**Results:**

In the participants with the TT genotype of FTO rs9939609 polymorphism, CRC risk was significantly associated with higher intake of dietary fat (OR:1.87 CI95%:1.76–1.99, *p* = 0.04), vitamin B3 (OR:1.20 CI95%:1.08–1.65, *p* = 0.04), and vitamin C (OR:1.06 CI95%:1.03–1.15, *p* = 0.04) and lower intake of β-carotene (OR:0.98 CI95%:0.97–0.99, *p* = 0.03), vitamin E (OR:0.77 CI95%:0.62–0.95, *p* = 0.02), vitamin B1 (OR:0.15 CI95%:0.04–0.50, *p* < 0.01), and biotin (OR:0.72 CI95%:0.0.57–0.92, *p* = 0.01). No significant association was found between CRC and dietary intake in carriers of AA/AT genotypes after adjustments for the confounders.

**Conclusion:**

CRC risk may be decreased by β-carotene, vitamins E, B1, and biotin only in those without the risk allele of the FTO gene. The association of CRC and diet may be influenced by FTO genotype. Further studies are warranted.

## Introduction

Adenomas or adenomatous polyps in the colon and rectum cause colorectal cancer (CRC) (1). Globally, CRC is ranked third (10%) and the second (9%) most common cancer, according to GLOBOCAN 202 (2, 3). There are 7 and 8 cases of CRC per 100,000 Iranians, respectively, which is the second and third most common cancers (4, 5). Until 2025, CRC will remain a malignant cancer with a growth rate of 54.1% (6).

Colorectal cancer is caused by a variety of factors, including demographic, genetic, lifestyle, and environmental factors. People with a family history of CRC are more likely to develop CRC than those under 50 (4). Non-hereditary CRCs are the most common types (7, 8) resulting from somatic mutations caused by lifestyle factors including drinking alcohol, smoking, not exercising, obesity, and eating a diet high in red meat and processed meats and low in fruits, vegetables, and calcium (6, 9–11). Obesity is one of the most important risk factors for CRC (12), which can be influenced both genetically and by lifestyle factors (13). In recent studies, it has been found that people with polymorphisms in genes that encode enzymes involved in nutrient metabolism may be at greater risk of CRC due to the excessive intake of calories and processed meat, which alters the level of expression of CRC-related genes (4).

CRC, as well as breast, pancreatic, and prostate cancers, are associated with the rs9939609 polymorphism of the fat mass and obesity-associated (FTO) gene (14–17). FTO is associated with food intake control, energy balance, and basal metabolic rate (BMR) (18); hypothalamic FTO gene expression was correlated with macronutrient intake (19, 20). Diet-related chronic diseases may be prevented by modifying FTO genotype in order to determine nutritional requirements (19). FTO rs9939609 polymorphism may influence both dietary intake and risk of colorectal cancer, according to some studies. African-Americans with the FTO rs9939609 polymorphism have a higher body mass index (BMI) and colorectal adenomas (21) Adipokines and FTO gene polymorphisms interact to promote colorectal cancer, according to Yamaji et al. Other studies have, however, failed to find a link between FTO rs9939609 polymorphism and CRC (22, 23). Dietary components may influence the association between FTO genotype and CRC. The polymorphisms in genes may alter the requirement for certain nutrients, thus preventing carcinogenesis. Nutrients may have a limited effect on CRC risk only among individuals who carry risk alleles in CRC genes. Therefore, the purpose of this study was to determine whether FTO gene polymorphisms and dietary intake interact in patients with CRC.

## Methods

### Study population

A case–control study was conducted on 450 randomly selected participants between January 2020 and June 2021, which included 150 CRC patients and 300 healthy individuals. Participants were referred to three hospitals (Firoozgar, Shohadaye Tajrish, and Taleghani) in Tehran, Iran between January 2020 and June 2021. Participants were selected based on their willingness to participate in this study, confirmation of histological CRC in their tissues, a minimum of 6 months after the first diagnosis of CRC, and age between 35 and 70 years old. Participants in the healthy control group had to be willing to take part in this study without malignancies and be between 35 and 70 years old. Among the exclusion criteria were inability to gather required data (*n* = 9), drugs affecting food intake (*n* = 2), or diseases associated with diabetes and fatty liver (*n* = 10). There were ultimately 429 participants (135 case and 294 control). In face-to-face interviews, data on demographic variables such as age, sex, marital status, and ethnicity were gathered.

### Ethical statement

The protocol of the study was approved by ethics committee of Shahid Beheshti University of Medical Sciences, Tehran, Iran (Code: IR.SBMU.CRC.REC.1398.028). The objectives of the study were explained verbally to all the patients and the control group and all participants signed the written consent form before participation in the study.

### Genotyping

A blood sample (5 mL) was collected from each participant in EDTA tubes (EDTA K3, Shandong Weigao Group Medical Polymer Co., Ltd., China) at the beginning of the study. Deoxyribonucleic acid (DNA) was extracted from 200 μL of whole blood using the salting out method. PCR amplification was performed using a PCR amplification instrument (GeneQ; Hangzhou Bioer Technology Co., Ltd., Hangzhou, China) and master mix DNA polymerase (cat. no A180301; Ampliqon, Denmark). To determine the genotype of the FTO gene rs9939609 polymorphism, the tetra-primer amplification refractory mutation system-polymerase chain reaction (T-ARMS-PCR) method was used. The sequences for the primers are presented in Table 1.

**Table 1:** The sequences of the primers used for tetra-primer amplification refractory mutation system-polymerase chain reaction (T-ARMS-PCR).

Gene	Primer sequence	Product size	Annealing temp. (°C)
FTO	Forward outer primer: AGTTCCAGTCATTTTTGACAGC Reverse outer primer: AGCCTCTCTACCATCTTATGTC Forward inner primer: CCTTGCGACTGCTGTGAATATA Reverse inner primer: GAGACTATCCAAGTGCATCTCA	Control fragment: 429 bp T allele: 278 bp A allele: 194 bp	59.5

### Physical activity assessment

Physical activity data were assessed using the International Physical Activity Questionnaire (IPAQ), which was previously validated in Iran (24). All IPAQ results were expressed and analyzed as metabolic equivalents per minute (MET-minutes per week).

### Dietary assessment

Dietary intake of the participants were assessed by a dietitian through a face-to-face interview (25). A validated 168-item semi-quantitative food frequency questionnaire (FFQ) was used included a list of foods with a standard serving size commonly consumed by Iranians. Participants were asked to report their frequency of consumption of a given serving of each food item during the previous year, on a daily (e.g., bread), weekly (e.g., meat) or monthly (e.g., fish) basis. Portion sizes of consumed foods were converted to grams per day using household measures (26). Then, the Nutritionist-IV software (First Databank Inc., Hearst Corp., San Bruno, CA) was used to analyze the intake of different types of dietary components including macronutrients and micronutrients. Each person’s daily calorie intake was calculated using the US Department of Agriculture food consumption database, which was modified for Iranian foods (27).

### Statistical analysis

An evaluation of genotype distribution was conducted using the Hardy–Weinberg equilibrium. Hardy–Weinberg equilibrium (HWE) is the state of the genotypic frequency of two alleles of one autosomal gene locus after one discrete generation of random mating in an indefinitely large population (28). Using chi-square and independent t-test methods, we compared general characteristics and the frequency of FTO rs9939609 polymorphisms between case and control groups. A binary logistic regression model based on the dominant genetic model (TT vs. AT+AA) was used to assess the association between CRC and the risk allele in different models including a crude model (Model 1), adjusted for age and sex (Model 2), adjusted for age, sex, physical activity, alcohol use, and smoking (Model 3), adjusted for age, sex, physical activity, alcohol use, smoking, calorie intake and BMI (Model 4). SPSS software version 21 (SPSS Inc., Chicago, United States) and *p*-value < 0.05 were used for all statistical analyses.

## Results

All measurements were normally distributed. The genotype distribution of the study population was in Hardy–Weinberg equilibrium. All of the participants had Persian ethnicity. Regarding *FTO rs9939609* genotype, about 36% (*n* = 154) of the participants had TT wild type genotype and 46% (*n* = 275) had AA/AT genotype. In people with TT genotype, the cases had higher BMI (27.67 ± 3.31 vs. 29.18 ± 3.9, *p* = 0.02) compared with the healthy controls. In the AA/AT group, the cases had higher age (52.5 ± 17.19 vs. 48.07 ± 11.19, *p* < 0.01), weight (69.62 ± 9.08 vs. 70.03 ± 10.89, *p* = 0.03) and BMI (27.58 ± 3.25 vs. 28.68 ± 4.06, *p* = 0.04) and lower height (158.65 ± 7.81 vs. 156.2 ± 5.71, *p* = 0.02). General characteristics of the participants according to the *FTO rs9939609* genotype are presented in Table 2.

**Table 2:** General characteristics of the case and control groups based on FTO rs9939609 genotype.

	TT genotype (*n* = 154)			AA/AT genotype (*n* = 275)			Cases (*n* = 46)	Controls (*n* = 108)	*p*	Cases (*n* = 89)	Controls (*n* = 186)	*p*
Age (years)	51.22 ± 16.08	47.37 ± 10.7	0.14	52.5 ± 17.19	48.07 ± 11.19	<0.01
Gender						
Males	25 (54.7%)	41 (47.6%)	0.08	58 (64.6%)	106 (57.3%)	0.2
Females	21 (45.3%)	57 (52.4%)		31 (35.4%)	80 (42.7%)	
Alcohol use	15 (33%)	5 (5%)	0.06	9 (10.5%)	14 (7.6%)	0.46
Smoking	3 (6%)	9 (8.6%)	0.59	37 (42%)	24 (13%)	0.005
Weight (kg)	70.85 ± 10.26	68.82 ± 7.86	0.18	70.03 ± 10.89	69.62 ± 9.08	0.03
Height (cm)	155.83 ± 4.72	157.83 ± 6.87	0.08	156.2 ± 5.71	158.65 ± 7.81	0.02
BMI (kg/m^2^)	29.18 ± 3.9	27.67 ± 3.31	0.02	28.68 ± 4.06	27.58 ± 3.25	0.04
physical activity (hrs/wk)	7.37 ± 1.84	7.30 ± 1.64	0.83	7.61 ± 2.02	7.33 ± 1.54	0.11

Regarding to the association of dietary intake and CRC, the cases with TT genotype of FTO rs9939609 polymorphism had lower intake of copper (1.49 ± 0.64 vs. 1.76 ± 0.71 g/d, *p* = 0.02), selenium (56.15 ± 22.97 vs. 67.26 ± 15.11 g/d, *p* < 0.01), β-carotene (2189.73 ± 474.3 vs. 2461.75 ± 772.57 g/d, *p* = 0.01), vitamin E (10.58 ± 4.14 vs. 13.99 ± 6.4 g/d, *p* < 0.01), tocopherol (8.46 ± 2.91 vs. 9.79 ± 4.53 g/d, *p* = 0.032), vitamin B_1_ (1.91 ± 0.87 vs. 2.3 ± 0.82 g/d, *p* = 0.01), folate (528 ± 0.61 vs. 574.39 ± 95.19 g/d, *p* = 0.01), biotin (26.76 ± 3.75 vs. 29.33 ± 6.61 g/d, *p* < 0.01) and higher intake of calorie (2500.48 ± 165.87 vs. 2594.64 ± 333.4 g/d, *p* = 0.021), fat (86.57 ± 10.38 vs. 93.25, ± 17.13 *p* < 0.01), fluoride (13967.59 ± 5662.25 vs. 11112.32 ± 3051.44 g/d, *p* < 0.01), vitamin A (819.7 ± 251.03 vs. 712.76 ± 113.86 g/d, *p* = 0.01), and vitamin K (157.9 ± 30.4 vs. 146.74 ± 21.64 g/d, *p* = 0.03). The cases with *rs9939609* AA/AT genotype had lower intake of selenium (49.74 ± 26.83 vs. 68.41 ± 16.53 g/d, *p* < 0.01) and folate (512.65 ± 99.73 vs. 566.94 ± 68.2, g/d *p* < 0.01), and higher intake of sodium (6465.85 ± 1840.38 vs. 6071.8 ± 1064.48 g/d, *p* < 0.01), fluoride (16708.7 ± 11263.98 vs. 10806.85 ± 3489.56 g/d, *p* < 0.01), chromium (0.12 ± 0.17 vs. 0.09 ± 0.15 g/d, *p* = 0.03), molybdenum (50.93 ± 10.29 vs. 50.88 ± 4.72 g/d, *p* < 0.01), vitamin A (850.94 ± 307.34 vs. 688.77 ± 172.6 g/d, *p* < 0.01), and vitamin K (162.51 ± 55.33 vs. 143.9 ± 27.75 g/d, *p* < 0.01) (Table 3).

**Table 3:** Dietary intake of the case and control groups based on FTO rs9939609 genotype.

	TT genotype			AA/AT genotype		
	Cases (*n* = 46)	Controls (*n* = 108)	*p*	Cases (*n* = 89)	Controls (*n* = 186)	*p*
Calorie (kcal/d)	2594.64 ± 333.4	2500.48 ± 165.87	0.02	2555.62 ± 449.78	2491.33 ± 182.2	0.46
Protein (g/d)	86.23 ± 13.6	86.14 ± 7.85	0.98	85.16 ± 23.3	85.43 ± 9.75	0.68
CHO (g/d)	369.47 ± 53.39	361.08 ± 28.88	0.21	367.88 ± 48.7	351.62 ± 34.95	0.95
Fat (g/d)	93.25 ± 17.13	86.57 ± 10.38	<0.01	89.84 ± 22.26	90.03 ± 10.64	0.17
Sodium (mg/d)	6144.12 ± 520.76	6108.42 ± 994.61	0.77	6465.85 ± 1840.38	6071.8 ± 1064.48	<0.01
Potassium (mg/d)	3920.35 ± 226.37	4066.28 ± 794.52	0.08	3928.21 ± 449.85	3969.28 ± 958.88	0.55
Iron (mg/d)	18.49 ± 2.7	18.69 ± 2.9	0.68	18.86 ± 3.44	18.54 ± 2.67	0.27
Calcium (mg/d)	1226.31 ± 53.03	1237.33 ± 228.90	0.64	1195.58 ± 140.29	1234.19 ± 622.43	0.58
Magnesium (mg/d)	335.79 ± 32.86	350.96 ± 65.54	0.06	330.7 ± 47.2	346.8 ± 71.2	0.68
Phosphorus (mg/d)	1403.16 ± 99.28	1422.52 ± 243.33	0.48	1391.25 ± 201.31	1411.49 ± 563.6	0.65
Zinc (mg/d)	10.5 ± 2.51	10.7 ± 3.17	0.67	10.86 ± 2.42	10.52 ± 3.62	0.30
Copper (μg/d)	1.49 ± 0.64	1.76 ± 0.71	0.02	1.61 ± 0.68	1.64 ± 0.67	0.89
Manganese (μg/d)	5.19 ± 1.95	5.18 ± 1.65	0.98	4.67 ± 1.62	5.2 ± 1.65	0.68
Selenium (μg/d)	56.15 ± 22.97	67.26 ± 15.11	<0.01	49.74 ± 26.83	68.41 ± 16.53	<0.01
Fluoride (μg/d)	13.59 ± 5.25	11.32 ± 3.44	<0.01	16.7 ± 11.98	10.85 ± 3.56	<0.01
Chromium (μg/d)	0.13 ± 0.18	0.1 ± 0.15	0.42	0.12 ± 0.17	0.09 ± 0.15	0.03
Molybdenum (μg/d)	50.81 ± 7.44	50.42 ± 4.72	0.74	50.93 ± 10.29	50.88 ± 4.72	<0.01
Vitamin A (IU/d)	819.7 ± 251.03	712.76 ± 113.86	0.01	850.94 ± 307.34	688.77 ± 172.6	<0.01
β-carotene (RE/d)	2189.73 ± 474.3	2461.75 ± 772.57	0.01	2034.09 ± 623.76	2482.07 ± 1058.26	0.26
Vitamin E (IU/d)	10.58 ± 4.14	13.99 ± 6.4	<0.01	10 ± 4.15	12.63 ± 4.66	0.31
Tocopherol (IU/d)	8.46 ± 2.91	9.79 ± 4.53	0.03	9.54 ± 5.81	9.64 ± 3.31	0.27
Vitamin B_1_ (mg/d)	1.91 ± 0.87	2.3 ± 0.82	0.01	2.06 ± 0.77	2.11 ± 0.86	0.48
Vitamin B_2_ (mg/d)	2.3 ± 1.06	2.4 ± 1.06	0.60	2.2 ± 0.96	2.3 ± 1.25	0.31
Vitamin B_3_ (mg/d)	21.59 ± 2.5	21.61 ± 2.9	0.96	21.39 ± 2.57	21.69 ± 3.05	0.17
Vitamin B_6_ (mg/d)	2.05 ± 0.83	1.82 ± 0.77	0.12	1.87 ± 0.79	1.99 ± 0.9	0.22
Folate (μg/d)	528 ± 0.61	574.39 ± 95.19	0.01	512.65 ± 99.73	566.94 ± 68.2	<0.01
Vitamin B_12_ (μg/d)	4.53 ± 1.67	4.31 ± 1.76	0.47	4.43 ± 2.06	4.33 ± 2.92	0.64
Pantothenic acid (μg/d)	5.13 ± 1.53	5.64 ± 1.65	0.07	5.71 ± 1.87	5.23 ± 2.08	0.90
Biotin (μg/d)	26.76 ± 3.75	29.33 ± 6.61	<0.01	27.02 ± 4.98	28.41 ± 8.5	0.31
Vitamin C (mg/d)	146.2 ± 22.4	154.33 ± 36.99	0.10	145.34 ± 20.56	151.11 ± 67.55	0.35
Vitamin D (μg/d)	1.14 ± 0.82	1.04 ± 0.77	0.51	0.99 ± 0.83	1.02 ± 0.78	0.84
Vitamin K (μg/d)	157.9 ± 30.4	146.74 ± 21.64	0.03	162.51 ± 55.33	143.9 ± 27.75	<0.01
Fiber (g/d)	23.99 ± 4.42	26.41 ± 7.63	0.01	23.77 ± 5.02	25.67 ± 4.62	0.26

Table 4 presents the results of logistic regression on the association between CRC and dietary intake among FTO *rs9939609* TT genotype carriers. The risk of CRC was positively associated with higher intake of dietary fat (OR:1.87 CI95%:1.76–1.99, *p* = 0.04), vitamin B3 (OR:1.20 CI95%:1.08–1.65, *p* = 0.04) and vitamin C (OR:1.06 CI95%:1.03–1.15, *p* = 0.04) and lower intake of β-carotene (OR:0.98, CI95%:0.97–0.99, *p* = 0.03), vitamin E (OR:0.77 CI95%:0.62–0.95, *p* = 0.02), vitamin B1 (OR:0.15 CI95%:0.04–0.50, *p* < 0.01), and biotin (OR:0.72 CI95%:0.0.57–0.92, *p* = 0.01) (Model 1). The results remained significant after adjustment for age and sex (Model 2). Further adjustments for physical activity, alcohol use, and smoking (Model 3) did not change the results. The association between CRC risk and higher intake of dietary fat (OR:1.65 CI95%:1.43–1.99, *p* = 0.04), vitamin B3 (OR:4.38 CI95%:1.06–18.12, *p* = 0.04) and vitamin C (OR:1.23 CI95%:1.01–1.50, *p* = 0.04) and lower intake of β-carotene (OR:0.97, CI95%:0.94–0.99, *p* = 0.04), vitamin E (OR:0.56, CI95%:0.35–0.89, *p* = 0.01), vitamin B1 (OR:0.11 CI95%:0.05–0.24, *p* = 0.01), and biotin (OR:0.30 CI95%:0.11–0.81, *p* = 0.02) remained significant after additional adjustments for calorie intake and BMI (Model 4). There was no significant association between CRC with the other nutrients in the carriers of TT genotype of FTO rs9939609 polymorphism.

**Table 4:** Logistic regression of the association between colorectal cancer and dietary intake among people with TT genotype of FTO rs9939609 polymorphism.

	Model 1			Model 2			Model 3			Model 4			OR	CI95%	*p*	OR	CI95%	*p*	OR	CI95%	*p*	OR	CI95%	*p*
Calorie (kcal/d)	1.00	0.92–1.02	0.79	0.99	0.96–1.02	0.56	0.99	0.96–0.02	0.46	0.99	0.95–1.02	0.49
Protein (g/d)	1.13	0.96–1.34	0.13	1.24	0.99–1.56	0.06	1.15	0.90–1.47	0.26	1.24	0.93–1.64	0.14
CHO (g/d)	0.98	0.92–1.04	0.56	1.00	0.92–1.09	0.98	0.98	0.90–1.08	0.74	0.97	0.87–1.08	0.61
Fat (g/d)	1.87	1.76–1.99	0.04	1.73	1.56--1.95	0.02	1.71	1.53–1.95	0.02	1.65	1.43–1.99	0.04
Sodium (mg/d)	0.99	0.98–1.01	0.24	0.98	0.96–1.00	0.08	0.98	0.96–0.99	0.05	0.98	0.96–1.00	0.05
Potassium (mg/d)	1.00	0.99–1.00	0.77	1.00	0.98–1.03	0.82	1.00	0.98–1.02	0.96	0.99	0.97–1.02	0.76
Iron (mg/d)	1.08	0.78–1.01	0.64	1.32	0.80–2.18	0.28	1.33	0.79–2.24	0.28	1.42	0.74–2.73	0.30
Calcium (mg/d)	1.01	0.99–1.03	0.46	1.02	0.99–1.06	0.13	1.03	0.99–1.06	0.13	1.03	0.99–1.07	0.17
Magnesium (mg/d)	1.01	0.96–1.08	0.61	1.05	0.96–1.14	0.28	1.08	0.97–1.20	0.14	1.14	0.98–1.34	0.09
Phosphorus (mg/d)	1.02	1.00–1.04	0.06	1.03	1.00–1.06	0.05	1.04	1.01–1.08	0.05	1.04	1.00–1.09	0.05
Zinc (mg/d)	0.94	0.72–1.23	0.65	1.01	0.68–1.51	0.95	0.84	0.52–1.35	0.47	0.65	0.33–1.32	0.23
Copper (μg/d)	0.75	0.27–2.08	0.58	0.52	0.12–2.31	0.39	0.21	0.03–1.66	0.14	0.35	0.04–3.28	0.36
Manganese (μg/d)	1.28	0.84–1.96	0.26	1.41	0.77–2.59	0.27	1.27	0.61–2.60	0.52	2.06	0.63–6.81	0.23
Selenium (μg/d)	1.08	0.97–1.19	0.15	1.16	0.99–1.35	0.06	1.19	1.01–1.42	0.05	1.13	0.93–1.36	0.22
Fluoride (μg/d)	1.00	1.00–1.01	0.13	1.01	1.00–1.02	0.13	1.011	1.00–1.02	0.11	1.01	1.00–1.02	0.13
Chromium (μg/d)	4.11	0.76–22.25	0.49	11.29	0.05–2.44	0.38	2.359	0.16–2.63	0.14	6.76	0.12–32.18	0.14
Molybdenum (μg/d)	0.93	0.78–1.11	0.42	0.87	0.69–1.09	0.22	0.85	0.65–1.11	0.24	0.73	0.46–1.14	0.17
Vitamin A (IU/d)	1.01	0.97–1.02	0.84	1.02	0.98–1.05	0.31	1.02	0.98–1.06	0.24	1.01	0.97–1.05	0.68
β-carotene (RE/d)	0.98	0.97–0.99	0.03	0.97	0.94–0.99	0.02	0.97	0.94–0.99	0.02	0.97	0.94–0.99	0.04
Vitamin E (IU/d)	0.77	0.62–0.95	0.02	0.68	0.51–0.92	0.01	0.61	0.43–0.87	0.01	0.56	0.35–0.89	0.01
Tocopherol (IU/d)	1.15	0.86–1.53	0.35	1.38	0.92–2.08	0.12	1.47	0.91–2.39	0.12	1.39	0.84–2.30	0.19
Vitamin B_1_ (mg/d)	0.15	0.04–0.50	<0.01	0.02	0.01–0.20	<0.01	0.01	0.01–0.15	<0.01	0.01	0.01–0.24	0.01
Vitamin B_2_ (mg/d)	0.58	0.26–1.16	0.12	0.47	0.18–1.21	0.12	0.37	0.13–1.09	0.07	0.27	0.06–1.23	0.09
Vitamin B_3_ (mg/d)	1.20	1.08–1.65	0.04	2.03	1.05–3.92	0.03	2.88	1.17–7.12	0.02	4.38	1.06–18.12	0.04
Vitamin B_6_ (mg/d)	1.95	0.74–5.11	0.17	2.50	0.67–9.22	0.17	3.48	0.84–4.44	0.08	4.89	0.86–27.58	0.07
Folate (μg/d)	1.03	0.97–1.08	0.38	1.06	0.97–1.15	0.22	1.06	0.97–1.15	0.18	1.05	0.96–1.15	0.27
Vitamin B_12_ (μg/d)	1.28	0.80–2.07	0.30	0.74	0.39–1.12	0.37	0.72	0.35–1.44	0.35	0.74	0.35–1.52	0.41
Pantothenic acid (μg/d)	0.58	0.33–1.02	0.14	0.33	0.15–0.74	0.01	0.25	0.09–0.70	0.01	0.18	0.04–0.80	0.02
Biotin (μg/d)	0.72	0.57–0.92	0.01	0.43	0.24–0.74	<0.01	0.36	0.18–0.70	<0.01	0.30	0.11–0.81	0.02
Vitamin C (mg/d)	1.06	1.03–1.15	0.04	1.13	1.00–1.28	0.04	1.18	1.01–1.38	0.03	1.23	1.01–1.50	0.04
Vitamin D (μg/d)	1.24	0.26–1.92	0.49	0.45	0.11–1.85	0.27	0.34	0.07–1.68	0.18	0.29	0.05–1.78	0.18
Vitamin K (μg/d)	1.02	0.94–1.11	0.59	0.95	0.85–1.07	0.39	0.89	0.78–1.04	0.14	0.93	0.79–1.08	0.33
Fiber (g/d)	0.99	0.79–1.24	0.91	1.02	0.77–1.35	0.89	0.96	0.70–1.33	0.82	0.94	0.67–1.34	0.74

Model 1: crude. Model 2: adjusted for age and sex. Model 3: adjusted for age, sex, physical activity, alcohol use, and smoking. Model 4: adjusted for age, sex, physical activity, alcohol use, smoking, calorie intake and BMI.

The results of logistic regression on the association between CRC and dietary intake among people with AA/AT FTO *rs9939609* genotype are presented in Table 5. Higher intake of chromium (OR:10.12 CI95%:1.20–85.18, *p* = 0.03) and pantothenic acid (OR:1.24 CI95%:1.01–1.52, *p* = 0.04) were associated with the higher risk of CRC. However, the associations were disappeared after adjustments for age and sex (Model 2). No significant association was found between CRC and dietary intake in carriers of AA/AT genotypes after further adjustments for physical activity, alcohol use, and smoking (Model 3), and additional adjustments for calorie intake and BMI.

**Table 5:** Logistic regression of the association between colorectal cancer and dietary intake among people with AA/AT FTO rs9939609 genotype.

	Model 1			Model 2			Model 3			Model 4			OR	CI95%	*p*	OR	CI95%	*p*	OR	CI95%	*p*	OR	CI95%	*p*
Calorie (kcal/d)	1.01	0.99–1.02	0.26	1.01	0.99–1.02	0.26	1.01	0.99–1.02	0.27	1.01	0.99–1.02	0.24
Protein (g/d)	0.95	0.89–1.01	0.12	0.95	0.89–1.01	0.12	0.95	0.89–1.01	0.10	0.96	0.89–1.02	0.15
CHO (g/d)	0.97	0.94–1.01	0.15	0.97	0.94–1.01	0.16	0.97	0.94–1.01	0.17	0.97	0.94–1.01	0.15
Fat (g/d)	0.99	0.93–1.04	0.63	0.98	0.93–1.04	0.58	0.99	0.93–1.04	0.66	0.99	0.93–1.05	0.69
Sodium (mg/d)	1.00	0.99–1.01	0.84	1.00	0.99–1.01	0.75	1.00	0.99–1.01	0.75	1.00	0.99–1.01	0.83
Potassium (mg/d)	1.00	0.99–1.01	0.66	1.00	0.99–1.01	0.63	1.00	0.99–1.01	0.60	1.00	0.99–1.01	0.50
Iron (mg/d)	1.0	0.88–1.24	0.63	1.05	0.88–1.24	0.61	1.04	0.85–1.24	0.65	1.04	0.87–1.24	0.66
Calcium (mg/d)	0.99	0.98–1.07	0.36	0.99	0.98–1.01	0.50	0.99	0.98–1.01	0.53	0.99	0.98–1.01	0.51
Magnesium (mg/d)	0.99	0.96–1.02	0.65	0.99	0.96–1.02	0.63	0.99	0.96–1.02	0.63	0.99	0.97–1.02	0.77
Phosphorus (mg/d)	1.01	0.99–1.01	0.15	1.00	0.99–1.02	0.41	1.00	0.99–1.02	0.43	1.01	0.99–1.02	0.33
Zinc (mg/d)	1.12	0.97–1.30	0.13	1.12	0.96–1.30	0.15	1.10	0.95–0.29	0.21	1.11	0.95–1.29	0.19
Copper (μg/d)	1.08	0.61–1.90	0.79	1.03	0.58–1.82	0.92	1.02	0.58–1.81	0.93	1.02	0.58–1.81	0.95
Manganese (μg/d)	0.81	0.66–1.01	0.06	0.81	0.65–1.01	0.05	0.81	0.65–1.01	0.06	0.79	0.63–0.99	0.05
Selenium (μg/d)	0.98	0.93–1.03	0.38	0.98	0.92–1.03	0.37	0.98	1.93–1.03	0.37	0.97	0.92–1.02	0.35
Fluoride (μg/d)	1.01	1.01–1.01	0.08	1.01	0.99–1.01	0.13	1.01	1.01–1.02	0.12	1.01	0.99–1.03	0.11
Chromium (μg/d)	10.12	1.20–85.18	0.03	7.09	0.80–62.61	0.08	7.43	0.84–65.96	0.07	8.36	0.91–76.36	0.06
Molybdenum (μg/d)	0.97	0.90–1.05	0.48	0.99	0.91–1.08	0.85	1.01	0.92–1.08	0.95	0.99	0.92–0.09	0.97
Vitamin A (IU/d)	1.01	0.99–1.02	0.19	1.01	0.99–1.02	0.28	1.1	0.99–1.02	0.28	1.01	0.99–1.02	0.24
β-carotene (RE/d)	0.96	0.94–0.99	0.04	0.99	0.98–1.00	0.10	0.99	0.98–1.00	0.11	0.99	0.98–1.01	0.09
Vitamin E (IU/d)	0.97	0.86–1.10	0.65	0.99	0.88–1.12	0.92	1.00	0.88–1.12	0.94	1.00	0.88–1.13	0.97
Tocopherol (IU/d)	1.04	0.91–1.19	0.52	1.05	1.05–1.20	0.51	1.04	0.91–0.19	0.54	1.04	0.91–1.20	0.58
Vitamin B_1_ (mg/d)	1.12	0.71–1.77	0.61	1.08	0.68–1.71	0.75	1.08	0.68–1.72	0.75	1.09	0.68–1.74	0.72
Vitamin B_2_ (mg/d)	1.08	0.76–1.55	0.66	1.02	0.71–1.48	0.90	1.02	0.71–1.48	0.91	1.01	0.69–1.46	0.97
Vitamin B_3_ (mg/d)	1.07	0.90–1.26	0.45	1.08	0.90–1.28	0.41	1.08	0.91–1.29	0.38	1.10	0.92–1.32	0.28
Vitamin B_6_ (mg/d)	0.80	0.52–1.22	0.30	0.86	0.56–1.32	0.48	0.86	0.56–1.33	0.51	0.88	0.51–1.36	0.57
Folate (μg/d)	0.99	0.97–1.01	0.36	0.99	0.96–1.01	0.31	0.99	0.96–1.01	0.31	0.98	0.96–1.01	0.32
Vitamin B_12_ (μg/d)	1.01	0.83–1.22	0.92	1.01	0.82–1.21	0.99	1.01	0.83–1.23	0.95	1.01	0.83–1.23	0.91
Pantothenic acid (μg/d)	1.24	1.01–1.52	0.04	1.22	0.98–1.51	0.07	1.24	1.00–1.54	0.05	1.23	0.99–1.54	0.06
Biotin (μg/d)	1.03	0.94–1.13	0.48	1.03	0.94–1.13	0.47	1.04	0.95–1.14	0.42	1.03	0.94–1.01	0.51
Vitamin C (mg/d)	0.98	0.94–1.02	0.27	0.98	0.94–1.13	0.27	0.98	0.94–1.01	0.25	0.98	0.94–1.01	0.22
Vitamin D (μg/d)	0.75	0.48–1.18	0.21	0.72	0.45–1.14	0.16	0.72	0.45–1.15	0.17	0.73	0.46–1.16	0.19
Vitamin K (μg/d)	0.99	0.96–1.04	0.90	1.01	0.95–1.04	0.84	0.99	0.95–1.04	0.84	0.99	0.95–1.04	0.80
Fiber (g/d)	0.99	0.90–1.11	0.94	1.02	0.91–1.40	0.74	1.02	0.91–1.14	0.71	1.02	0.91–1.14	0.75

Model 1: crude. Model 2: adjusted for age and sex. Model 3: adjusted for age, sex, physical activity, alcohol use, and smoking. Model 4: adjusted for age, sex, physical activity, alcohol use, smoking, calorie intake and BMI.

## Discussion

As far as the authors know, this is the first study to explore the interaction between the risk allele of the FTO rs9939609 polymorphism and dietary intake of different types of nutrients in CRC patients. Participants with the TT genotype had a significantly higher risk of CRC when they had higher intake of fat, vitamin B3, and vitamin C and lower intake of β-carotene, vitamin E, vitamin B1, and biotin. A higher intake of chromium, pantothenic acid, and lower intake of β-carotene was associated with the higher CRC risk among patients carrying the AA/AT genotype. However, no significant association was found between CRC risk and nutrients in carriers of FTO gene risk allele (A) after adjustments for the confounders.

The effects of dietary components on the risk of CRC are frequently reported (29–31). The results of this study on the association of CRC and dietary fat was in line with some other studies (32, 33). For example, one study reported a possible association between CRC with total fat, polyunsaturated fatty acids, and trans fatty acids (34). In line with the results of the present study, recent studies indicated that the association between CRC risk and dietary components can be influenced by FTO genotype (35, 36). For example, a case–control study found an inverse association between CRC and total dietary fiber intake only among people with AA/AT FTO rs9939609 genotype (35). Previous studies (37–40) have shown that dietary fiber can reduce the risk of CRC by promoting fermentation by gut bacteria as a prebiotic and producing short chain fatty acids (SCFAs) (37). Butyrate, a type of SCFA, has been found to inhibit neuropilin-1, a receptor commonly found in CRC cells (41). Additionally, butyrate may induce apoptosis and suppress the proliferation and invasion of CRC cells by regulating microRNAs such as miR-92a and miR-203 (42, 43). Butyrate may also inhibit the motility of CRC cells by blocking the Akt/ERK signaling pathway, suggesting its potential in preventing metastatic CRC (44).

In current study, there was an interaction between some micronutrients and the FTO rs9939609 genotype regarding CRC risk. Higher intake of dietary fat, vitamin B3, and vitamin C intake may increase the risk of CRC, whereas vitamin E, vitamin B1, and biotin intake may reduce the risk only among carriers of the FTO SNP rs9939609. Regarding the association of CRC with vitamin B3, a recent study found that niacin inhibits tumor necrosis factor-related apoptosis-inducing ligand (TRAIL)-induced apoptosis through activation of autophagic flux in human CRC cells (45). Using the HCT116 human colon carcinoma cell line, this study showed that niacin activates autophagy, and that the autophagy activation protects tumor cells from TRAIL-induced mitochondrial membrane dysfunction and tumor cell death. Interestingly, one recent study reported vitamin C and niacin have a dose-dependent effect on CRC risk. High doses of vitamin C and niacin (30 mg/kg body weight) killed CRC stem cells, whereas low doses induced proliferation of HT-29 and HCT-15 CRC stem cells (46). So, the effects of niacin and vitamin C vitamins on CRC risk may be influenced by FTO polymorphism due to the possible effects of the FTO gene polymorphisms on the level of the requirements to these vitamins (47, 48).

According to the present study, carriers of both TT and AT and AA genotypes of rs9939609 polymorphism may be protected against CRC by higher intakes of carriers. The protective antioxidant effects of carotenoids found in fruits and vegetables are frequently reported (49–52). In line with this study, previous studies reported that β-carotene intake was inversely associated with CRC risk (53–55). A cohort study found an inverse association between the risk of CRC and β-carotene intake only in male current smokers (56). A possible mechanism for the chemo-preventive and antiproliferative effects of carotenoid in cancer cells is downregulation of COX-2 but not that of COX-1 in LS-174 cells, indicating that this is a specific effect in either transcriptional activity or in RNA stability. In addition, the carotenoid was able to downregulate baseline and heregulin-α-induced expression of COX-2 and PGE2 content in CRC cells (57).

A higher intake of pantothenic acid was associated with a lower risk of CRC among TT genotype carriers of the FTO SNP rs9939609, while with a higher risk of CRC among A allele carriers of the FTO SNP rs9939609. The body uses pantothenic acid to utilize carbohydrates, proteins, and lipids. Pantothenic acid plays a key role as a cofactor for the synthesis of CoA (58) which is required for the tricarboxylic acid cycle, fatty acid metabolism, and acylation reactions. As a result of CoA catabolism, pantothenate and cysteamine are produced, the latter of which potentiates inflammation. This chemical breaks down disulfide bonds, inactivating proteins and directly inhibiting glutathione synthase. During inflammation, cysteamine is thought to generate oxidative stress (ROS) (59). However, the role of pantothenic acid on the risk of CRC have not yet been examined. The availability of pantothenic acid may modulate tumorigenesis according to epidemiological and laboratory animal studies (59). Genetic polymorphisms and their impact on dietary requirements determine whether nutrients are carcinogenic or anti-carcinogenic (60).

The strengths of the present study are the large number of the participants and adjustment for potential confounding factors, including age, sex, physical activity, alcohol use, smoking, calorie intake and BMI. Moreover, to our knowledge, this is the first study to investigate the effects of FTO gene rs9939609 polymorphism on the association between CRC and dietary components. However, this study had some limitations. The FFQ which was used for dietary assessment is a self-reporting tool and may contain measuring errors such as underreporting of dietary intake in obese and overweight participants. Also, the interactions between CRC, FTO gene, and diet were only assessed in the dominant genetic model and it was not possible to determine the effect of the number of risk alleles on the relationship between dietary intake and CRC in patients with CRC due to the limited study sample size. Future longitudinal studies with larger sample size of different FTO genotypes are needed to explore the exact interactions between CRC, FTO gene, and dietary intake.

## Conclusion

The results of the present study showed that the FTO gene genotype is significantly effective on the relationship between diet and CRC. Some nutrients including dietary fat, vitamin B3, and vitamin C may act as risk factors and some other nutrients including β-carotene, vitamin E, vitamin B1, and biotin may be protective against the risk of CRC only in people without the risk allele of the FTO gene. There is a need for future longitudinal research to understand the mutual effects of dietary factors and FTO gene polymorphisms on the risk of CRC.

## Data availability statement

The raw data supporting the conclusions of this article will be made available by the authors, without undue reservation.

## Ethics statement

The studies involving human participants were reviewed and approved by: IR.SBMU.CRC.REC.1398.028. The patients/participants provided their written informed consent to participate in this study.

## Author contributions

SD, MGH, MJ, KhAM, AA, FA, NV, SAA, MEA, JP, GHAT and SAMJ designed the study, and were involved in the data collection, analysis, and drafting of the manuscript. MKM, MR, MA, HS, PH, SMD, MB and SD were involved in the design of the study, analysis of the data, and critically reviewed the manuscript. All authors read and approved the final manuscript.

## Funding

Funding for this study was provided by Shahid Beheshti University of Medical Sciences, Tehran, Iran (Code 15784).

## Conflict of interest

The authors declare that the research was conducted in the absence of any commercial or financial relationships that could be construed as a potential conflict of interest.

## Publisher’s note

All claims expressed in this article are solely those of the authors and do not necessarily represent those of their affiliated organizations, or those of the publisher, the editors and the reviewers. Any product that may be evaluated in this article, or claim that may be made by its manufacturer, is not guaranteed or endorsed by the publisher.
